# Supplementary material for: Kagome van-der-Waals Pd3P2S8 with flat band
Source: Sci Rep. 2020 Dec 2;10:20998. doi: 10.1038/s41598-020-77825-1 (PMC7710707; doi:10.1038/s41598-020-77825-1)
Supplement: Supplementary file 1 — Supplementary Information. [file 41598_2020_77825_MOESM1_ESM.pdf]

Kagome van-der-Waals $Pd_3P_2S_8$ with flat band

Seunghyun Park[1,2], Soonmin Kang[1], Haeri Kim[1], Ki Hoon Lee[1], Pilkwang Kim[2], Sangwoo Sim[1,2], Nahyun Lee[1], Balamurugan Karuppannan[1], Junghyun Kim[1], Jonghyeon Kim[3], Kyung Ik Sim[3], Matthew J. Coak[1], Yukio Noda[4], Cheol-Hwan Park[2], Jae Hoon Kim[3] and Je-Geun Park[1,2,5,*]

[1]Center for Correlated Electron Systems, Institute for Basic Science (IBS-CCES), Seoul, 08826, Republic of Korea
[2]Department of Physics and Astronomy, Seoul National University, Seoul, 08826, Republic of Korea
[3]Department of Physics, Yonsei University, Seoul, 03722, Republic of Korea
[4]Institute of Multidisciplinary Research for Advanced Materials, Tohoku University, Sendai, 980-8577, Japan
[5]Center for Quantum Materials, Seoul National University, Seoul, 08826, Republic of Korea
[*]jgpark10@snu.ac.kr

Supplementary Information

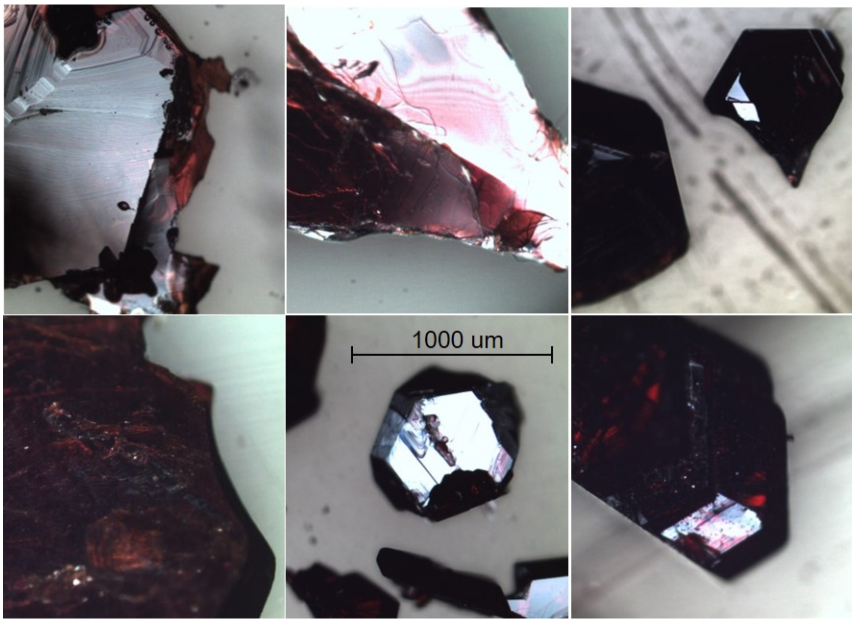

Supplementary Figure S1. The optical image of the samples. Each crystals' quality was confirmed by EDX and XRD. The magnetization measurement was done with all of the samples. Samples from left to right, top to bottom are respectively labeled as Sample 1808, 15,

17, 1310, 1805, and 1807. Sample 1808 was synthesized by the method identical to Zhang et al.[55] The others were synthesized in another method described in the experimental section.

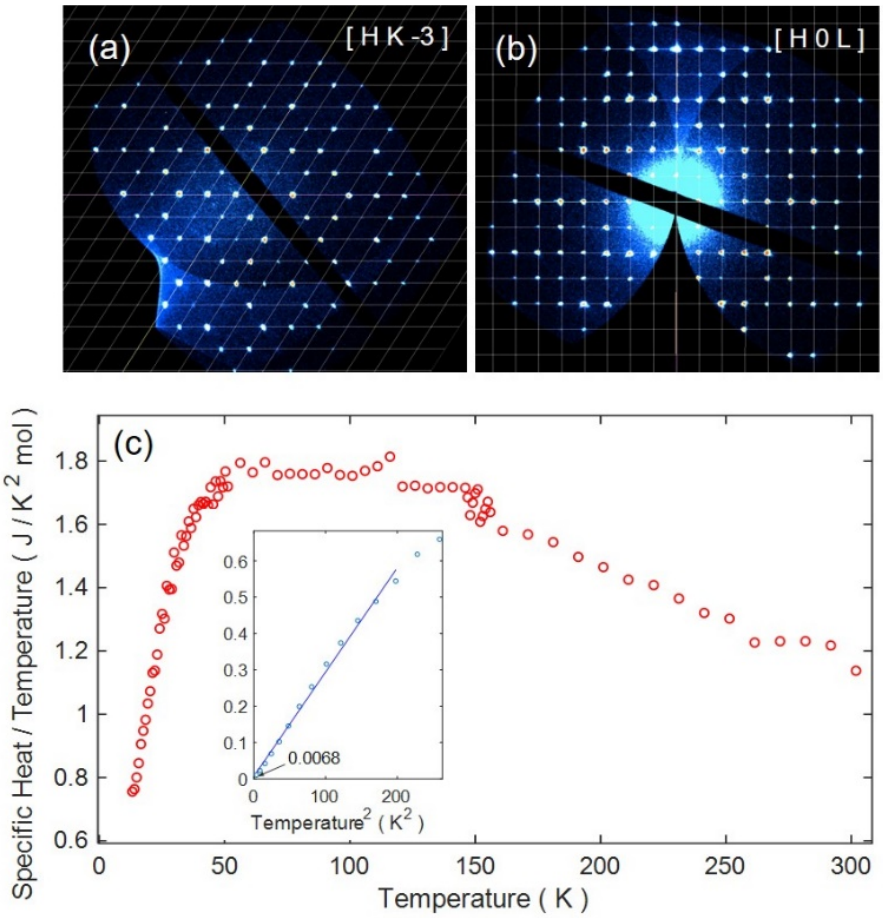

Supplementary Figure S2. a, b) Single-crystal XRD of the sample. The diffraction pattern fits the triangular symmetry for (h k l) = (h k -3) and (h k l) = (h 0 1), consistent with the space group of P-3m1. c) The specific heat data divided by temperature is presented as a function of temperature. There is no significant peak to suggest a possible phase transition. (insert) The specific heat data divided by temperature are shown as a function of the square of temperature. By fitting the low-temperature data, one can obtain the following values: γ = 0.0068 J / K^4 mol and β = 0.0029 J / K^2 mol. The former is the fermionic contribution, while the latter is typical behavior of phonon contribution with an estimated Debye temperature of about 200 K.

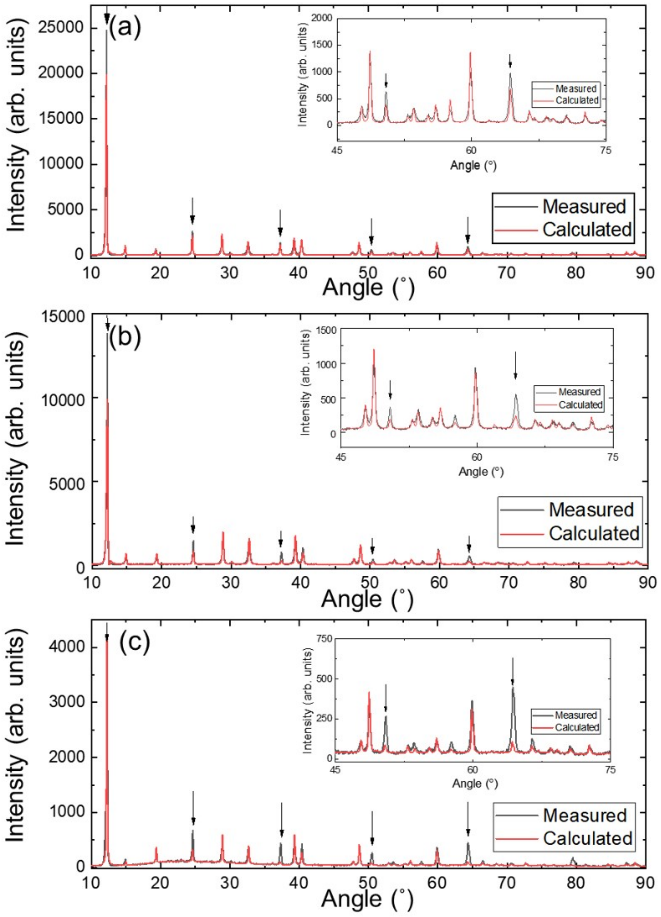

Supplementary Figure S3. a, b, c) is the powder XRD data of samples 1505, 17, and 1807, respectively. The arrows indicate the (001) peaks of the space group of P-3m1. The preferred orientation of the [001] direction is present due to the van-der-Waals structure, which occurs by pressing the material with the glass slide for powder XRD. The peaks have an identical position compared to the fitter peaks, and agreeable relative intensities. The absence of any additional impurity peaks emphasizes the excellent quality of the sample.

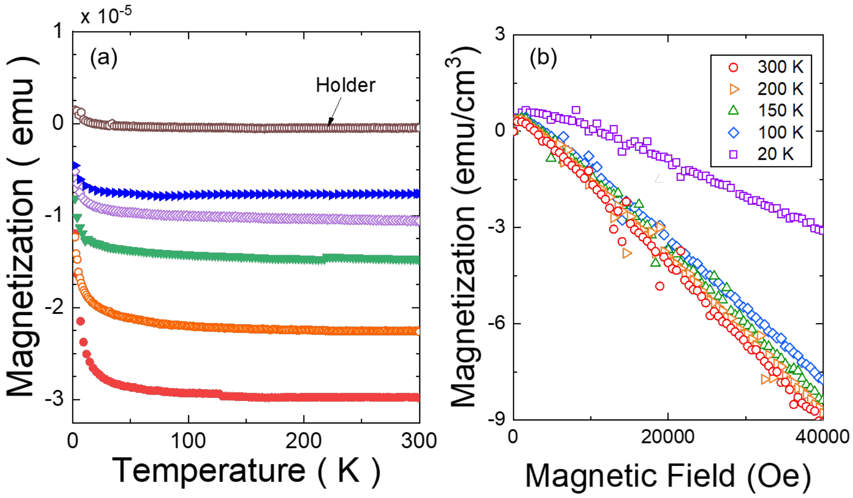

Supplementary Figure S4. a) The raw data of the magnetization measurement as a function of temperature under 1 T of the applied magnetic field. The brown graph with the negligible signals of at most ~ 5×10^{-7} emu is those of the quartz holder and GE varnish. b) Molar magnetization data in the temperature range of 20 to 300 K. The slope of each graph, which corresponds to the susceptibility, matches with the value obtained from the temperature sweep.

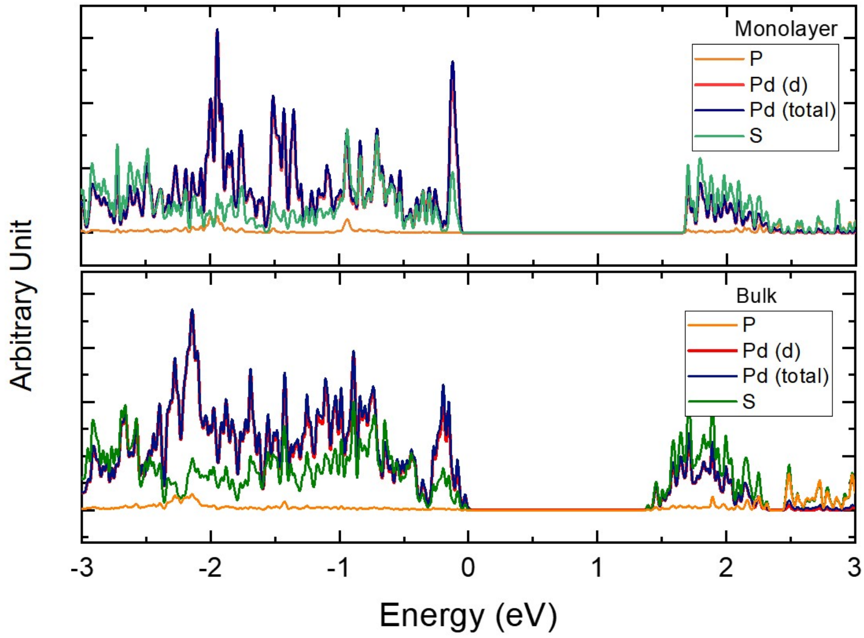

Supplementary Figure S5. Contribution of the Pd *d*-orbital to the calculated electronic density of states. One can see that the flat kagome band at the top of the valence band is composed mostly of the Pd *d*-orbitals. The dominant character is preserved down to the monolayer.

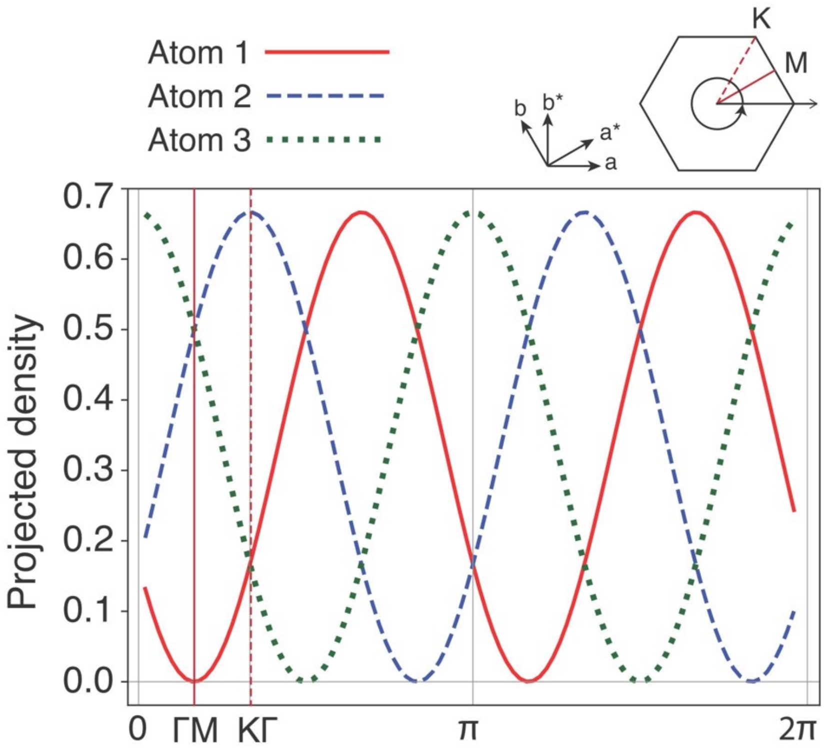

Supplementary Figure S6. The contribution of orbitals at each atomic site as a function of the azimuthal angle around the Brillouin zone center. Here, we chose the radius of the circle to be 7.5 % of the ΓK direction. It is possible to observe the vanishing contribution of atom 1 in the ΓM direction.

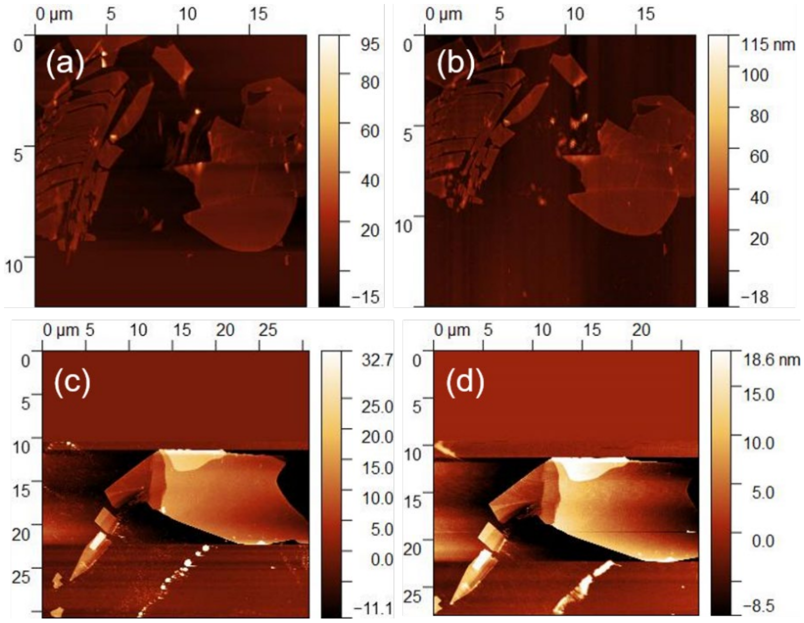

Supplementary Figure S7. AFM images of flakes that are exposed in a, b) ethanol and c, d) water, respectively, for 25 hours. The thickness of the flakes a, c) before and b, d) after the exposure was measured by AFM. No change in the thickness was detected. The thickness of the flakes vary from a, b) 10 to 15 nm, and c, d) 5 to 30 nm.
